# 1-(4-{[(*E*)-4-Methyl­benzyl­idene]amino}­phen­yl)ethanone oxime

**DOI:** 10.1107/S1600536810034598

**Published:** 2010-09-04

**Authors:** Li Zhao, Seik Weng Ng

**Affiliations:** a School of Chemical and Biological Engineering, Lanzhou Jiaotong University, Lanzhou 730070, People’s Republic of China; b Department of Chemistry, University of Malaya, 50603 Kuala Lumpur, Malaysia

## Abstract

In the title compound, C_16_H_16_N_2_O, the dihedral angle formed by the two benzene rings is 50.3 (1)°. In the crystal structure, mol­ecules are linked into an infinite one-dimensional supra­molecular structure by inter­molecular O—H⋯N hydrogen-bond inter­actions.

## Related literature

For background to oxime-type compounds, see: Dong *et al.* (2009*a* ▶,*b* ▶). For the synthesis, see: Rafiq *et al.* (2008 ▶); Dong *et al.* (2009*c* ▶).
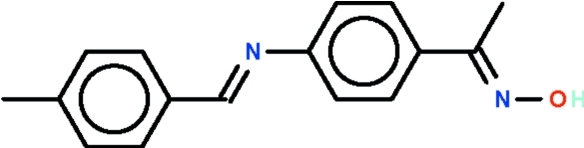


## Experimental

### 

#### Crystal data

C_16_H_16_N_2_O
*M* *_r_* = 252.31
Monoclinic, 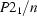

*a* = 5.7785 (6) Å
*b* = 14.581 (2) Å
*c* = 16.226 (2) Å
β = 94.285 (1)°
*V* = 1363.4 (2) Å^3^
*Z* = 4
Mo *K*α radiation
μ = 0.08 mm^−1^
*T* = 293 K
0.45 × 0.15 × 0.10 mm

#### Data collection

Bruker SMART diffractometer
Absorption correction: multi-scan (*SADABS*; Sheldrick, 1996 ▶) *T*_min_ = 0.966, *T*_max_ = 0.992
6860 measured reflections
2396 independent reflections
1480 reflections with *I* > 2σ(*I*)
*R*_int_ = 0.054

#### Refinement

*R*[*F* ^2^ > 2σ(*F* ^2^)] = 0.047
*wR*(*F* ^2^) = 0.136
*S* = 0.95
2396 reflections
178 parameters
1 restraint
H atoms treated by a mixture of independent and constrained refinement
Δρ_max_ = 0.15 e Å^−3^
Δρ_min_ = −0.17 e Å^−3^

### 

Data collection: *SMART* (Bruker, 1996 ▶); cell refinement: *SAINT* (Bruker, 1996 ▶); data reduction: *SAINT*; program(s) used to solve structure: *SHELXS97* (Sheldrick, 2008 ▶); program(s) used to refine structure: *SHELXL97* (Sheldrick, 2008 ▶); molecular graphics: *X-SEED* (Barbour, 2001 ▶); software used to prepare material for publication: *publCIF* (Westrip, 2010 ▶).

## Supplementary Material

Crystal structure: contains datablocks global, I. DOI: 10.1107/S1600536810034598/hg2705sup1.cif

Structure factors: contains datablocks I. DOI: 10.1107/S1600536810034598/hg2705Isup2.hkl

Additional supplementary materials:  crystallographic information; 3D view; checkCIF report

## Figures and Tables

**Table 1:** Hydrogen-bond geometry (Å, °)

*D*—H⋯*A*	*D*—H	H⋯*A*	*D*⋯*A*	*D*—H⋯*A*
O1—H1⋯N2^i^	0.86 (1)	2.06 (1)	2.919 (2)	175 (3)

Symmetry code: (i) 
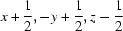
.

**Fig. 1.**
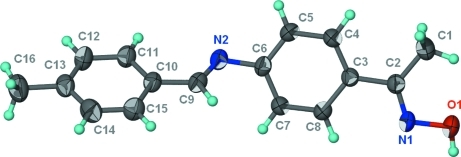
Thermal ellipsoid plot (Barbour, 2001) of C16H16N2O at the 50% probability level; hydrogen atoms are drawn as spheres of arbitrary radius.

**Fig. 2.**
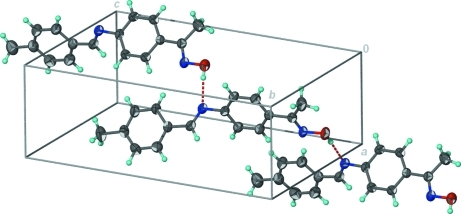
Part of one-dimensional supramolecular structure is formed by O—H···N intermolecular interaction with H bonds drawn as dotted lines.

## supplementary crystallographic information

### Comment

Oxime-type compounds are a great important ligands in modern coordination chemistry (Dong *et al.*, 2009*a*; Dong *et al.*, 2009*b*). Structures of oxime-type compounds derived from substituted benzaldehydes and 1-(4-aminophenyl)ethanone haven't been reported so far (Rafiq *et al.*, 2008). Here we report the synthesis and crystal structure of (*E*)-4-[1-(Hydroxyimino)ethyl]-*N*-(4-methylbenzylidene)aniline (I), (Fig. 1).

The single-crystal structure of the title compound is built up by discrete C_16_H_16_N_2_O molecules, in which all bond lengths are in normal ranges. Within the molecule, the dihedral angle formed by the two benzene rings is 50.3 (1)°. In the crystal structure, the molecules are linked into infinite one-dimensional supramolecular structure by intermolecular O—H···N hydrogen bond interaction (Table 1 and Fig. 2).

### Experimental

4-Aminophenylethanone oxime was prepared by 1-(4-aminophenyl)ethanone, hydroxylamine sulfate and sodium acetate (Rafiq *et al.*, 2008; Dong *et al.*, 2009*c*). To an ethanol solution (7 ml) of 4-aminophenylethanone oxime (151.0 mg, 1.00 mmol) was added dropwise an ethanol solution (8 ml) of 4-methylbenzaldehyde (121.6 mg, 1.00 mmol). The mixture solution was stirred at 330 K for 4 h. After cooling to room temperature, the precipitate was filtered off, and washed successively three times with ethanol. The product was dried *in vacuo* and purified by recrystallization from ethanol to yield 220.3 mg (Yield, 80.8%) of solid; m.p. 471–472 K. Pale-yellow block-like single crystals suitable for X-ray diffraction studies were obtained by slow evaporation from a solution of acetone of (I) at room temperature for about two weeks. Anal. Calcd. for C_16_H_16_N_2_O: C, 76.16; H, 6.39; N, 11.10; Found: C, 76.08; H, 6.45; N, 11.02.

### Refinement

Carbon-bound H-atoms were placed in calculated positions (C—H 0.93 to 0.96 Å) and were included in the refinement in the riding model approximation, with U(H) set to 1.2 to 1.5U(C).

The hydroxy H-atom was located in a difference Fourier map, and was refined with a distance restraint of O–H of 0.85±0.01 Å; its temperature factor was freely refined.

### Crystal data

**Table:**

C_16_H_16_N_2_O	*F*(000) = 536
*M**_r_* = 252.31	*D*_x_ = 1.229 Mg m^−^^3^
Monoclinic, *P*2_1_/*n*	Mo *K*α radiation, λ = 0.71073 Å
Hall symbol: -P 2yn	Cell parameters from 1735 reflections
*a* = 5.7785 (6) Å	θ = 2.5–25.3°
*b* = 14.581 (2) Å	µ = 0.08 mm^−^^1^
*c* = 16.226 (2) Å	*T* = 293 K
β = 94.285 (1)°	Block, yellow
*V* = 1363.4 (2) Å^3^	0.45 × 0.15 × 0.10 mm
*Z* = 4	

### Data collection

**Table:**

Bruker SMART diffractometer	2396 independent reflections
Radiation source: fine-focus sealed tube	1480 reflections with *I* > 2σ(*I*)
graphite	*R*_int_ = 0.054
φ and ω scans	θ_max_ = 25.0°, θ_min_ = 1.9°
Absorption correction: multi-scan (*SADABS*; Sheldrick, 1996)	*h* = −6→6
*T*_min_ = 0.966, *T*_max_ = 0.992	*k* = −17→14
6860 measured reflections	*l* = −18→19

### Refinement

**Table:**

Refinement on *F*^2^	Primary atom site location: structure-invariant direct methods
Least-squares matrix: full	Secondary atom site location: difference Fourier map
*R*[*F*^2^ > 2σ(*F*^2^)] = 0.047	Hydrogen site location: inferred from neighbouring sites
*wR*(*F*^2^) = 0.136	H atoms treated by a mixture of independent and constrained refinement
*S* = 0.95	*w* = 1/[σ^2^(*F*_o_^2^) + (0.0748*P*)^2^] where *P* = (*F*_o_^2^ + 2*F*_c_^2^)/3
2396 reflections	(Δ/σ)_max_ = 0.001
178 parameters	Δρ_max_ = 0.15 e Å^−^^3^
1 restraint	Δρ_min_ = −0.17 e Å^−^^3^

### Special details

**Table:**

Geometry. All e.s.d.'s (except the e.s.d. in the dihedral angle between two l.s. planes) are estimated using the full covariance matrix. The cell e.s.d.'s are taken into account individually in the estimation of e.s.d.'s in distances, angles and torsion angles; correlations between e.s.d.'s in cell parameters are only used when they are defined by crystal symmetry. An approximate (isotropic) treatment of cell e.s.d.'s is used for estimating e.s.d.'s involving l.s. planes.

### Fractional atomic coordinates and isotropic or equivalent isotropic displacement parameters (Å^2^)

**Table:**

	*x*	*y*	*z*	*U*_iso_*/*U*_eq_	
O1	0.7517 (3)	0.26556 (12)	0.05236 (8)	0.0674 (5)	
H1	0.861 (4)	0.2274 (16)	0.0445 (16)	0.107 (11)*	
N1	0.7670 (3)	0.27459 (12)	0.13916 (9)	0.0529 (5)	
N2	0.6350 (3)	0.35564 (11)	0.51824 (10)	0.0512 (5)	
C1	0.4032 (4)	0.36066 (17)	0.11219 (13)	0.0710 (7)	
H1A	0.4313	0.3513	0.0552	0.106*	
H1B	0.3934	0.4252	0.1231	0.106*	
H1C	0.2599	0.3316	0.1236	0.106*	
C2	0.5974 (4)	0.32006 (13)	0.16606 (11)	0.0457 (5)	
C3	0.6050 (3)	0.32942 (12)	0.25745 (11)	0.0419 (5)	
C4	0.4248 (4)	0.36780 (14)	0.29717 (12)	0.0515 (5)	
H4	0.2943	0.3890	0.2659	0.062*	
C5	0.4343 (4)	0.37537 (14)	0.38259 (12)	0.0531 (6)	
H5	0.3091	0.4001	0.4078	0.064*	
C6	0.6290 (4)	0.34636 (13)	0.43064 (11)	0.0451 (5)	
C7	0.8106 (4)	0.30705 (14)	0.39156 (11)	0.0508 (5)	
H7	0.9415	0.2861	0.4228	0.061*	
C8	0.7974 (4)	0.29896 (13)	0.30692 (12)	0.0506 (5)	
H8	0.9206	0.2724	0.2819	0.061*	
C9	0.8175 (4)	0.39055 (13)	0.55448 (12)	0.0502 (5)	
H9	0.9316	0.4104	0.5211	0.060*	
C10	0.8639 (4)	0.40224 (12)	0.64333 (12)	0.0470 (5)	
C11	0.7108 (4)	0.37421 (14)	0.70052 (12)	0.0554 (6)	
H11	0.5694	0.3481	0.6823	0.066*	
C12	0.7675 (4)	0.38494 (14)	0.78433 (12)	0.0605 (6)	
H12	0.6630	0.3658	0.8217	0.073*	
C13	0.9766 (4)	0.42353 (14)	0.81375 (13)	0.0555 (6)	
C14	1.1268 (4)	0.45209 (14)	0.75684 (13)	0.0624 (6)	
H14	1.2677	0.4786	0.7752	0.075*	
C15	1.0720 (4)	0.44209 (14)	0.67304 (13)	0.0593 (6)	
H15	1.1759	0.4623	0.6359	0.071*	
C16	1.0399 (5)	0.43398 (18)	0.90515 (13)	0.0821 (8)	
H16A	1.1710	0.3958	0.9210	0.123*	
H16B	0.9106	0.4160	0.9354	0.123*	
H16C	1.0783	0.4968	0.9173	0.123*	

### Atomic displacement parameters (Å^2^)

**Table:**

	*U*^11^	*U*^22^	*U*^33^	*U*^12^	*U*^13^	*U*^23^
O1	0.0689 (12)	0.0968 (13)	0.0364 (9)	0.0112 (10)	0.0044 (7)	−0.0072 (8)
N1	0.0546 (12)	0.0723 (12)	0.0319 (9)	0.0032 (9)	0.0043 (8)	−0.0032 (8)
N2	0.0532 (11)	0.0621 (11)	0.0388 (10)	−0.0012 (9)	0.0078 (8)	−0.0002 (8)
C1	0.0772 (18)	0.0874 (17)	0.0466 (13)	0.0216 (14)	−0.0075 (12)	−0.0056 (11)
C2	0.0468 (13)	0.0492 (12)	0.0411 (11)	−0.0013 (10)	0.0027 (9)	0.0008 (9)
C3	0.0436 (12)	0.0441 (11)	0.0383 (10)	−0.0009 (9)	0.0034 (9)	0.0033 (8)
C4	0.0461 (13)	0.0631 (13)	0.0449 (12)	0.0082 (10)	0.0015 (10)	0.0029 (9)
C5	0.0472 (13)	0.0670 (14)	0.0462 (12)	0.0069 (11)	0.0110 (10)	−0.0012 (9)
C6	0.0503 (13)	0.0503 (11)	0.0356 (11)	−0.0028 (9)	0.0078 (9)	0.0025 (8)
C7	0.0469 (13)	0.0632 (13)	0.0422 (12)	0.0074 (10)	0.0016 (10)	0.0018 (9)
C8	0.0481 (13)	0.0610 (13)	0.0430 (12)	0.0088 (10)	0.0065 (10)	−0.0019 (9)
C9	0.0543 (14)	0.0529 (12)	0.0447 (12)	−0.0008 (10)	0.0122 (10)	0.0012 (9)
C10	0.0556 (14)	0.0451 (11)	0.0406 (11)	0.0013 (10)	0.0050 (10)	−0.0027 (8)
C11	0.0566 (14)	0.0648 (13)	0.0448 (12)	−0.0067 (11)	0.0048 (10)	−0.0059 (10)
C12	0.0717 (16)	0.0685 (15)	0.0419 (12)	−0.0052 (12)	0.0078 (11)	−0.0055 (10)
C13	0.0699 (16)	0.0499 (12)	0.0459 (12)	0.0042 (11)	−0.0017 (11)	−0.0061 (9)
C14	0.0628 (16)	0.0594 (13)	0.0629 (15)	−0.0095 (11)	−0.0096 (12)	−0.0083 (11)
C15	0.0609 (15)	0.0594 (14)	0.0582 (14)	−0.0098 (11)	0.0084 (12)	−0.0013 (10)
C16	0.101 (2)	0.0889 (18)	0.0538 (15)	0.0072 (15)	−0.0125 (14)	−0.0117 (12)

### Geometric parameters (Å, °)

**Table:**

O1—N1	1.4107 (19)	C7—H7	0.9300
O1—H1	0.860 (10)	C8—H8	0.9300
N1—C2	1.286 (2)	C9—C10	1.457 (3)
N2—C9	1.275 (2)	C9—H9	0.9300
N2—C6	1.426 (2)	C10—C15	1.389 (3)
C1—C2	1.492 (3)	C10—C11	1.390 (3)
C1—H1A	0.9600	C11—C12	1.384 (3)
C1—H1B	0.9600	C11—H11	0.9300
C1—H1C	0.9600	C12—C13	1.385 (3)
C2—C3	1.487 (2)	C12—H12	0.9300
C3—C4	1.383 (3)	C13—C14	1.378 (3)
C3—C8	1.394 (3)	C13—C16	1.509 (3)
C4—C5	1.387 (3)	C14—C15	1.381 (3)
C4—H4	0.9300	C14—H14	0.9300
C5—C6	1.386 (3)	C15—H15	0.9300
C5—H5	0.9300	C16—H16A	0.9600
C6—C7	1.389 (3)	C16—H16B	0.9600
C7—C8	1.375 (3)	C16—H16C	0.9600
			
N1—O1—H1	102.5 (18)	C3—C8—H8	119.0
C2—N1—O1	113.22 (16)	N2—C9—C10	126.0 (2)
C9—N2—C6	117.10 (18)	N2—C9—H9	117.0
C2—C1—H1A	109.5	C10—C9—H9	117.0
C2—C1—H1B	109.5	C15—C10—C11	117.95 (19)
H1A—C1—H1B	109.5	C15—C10—C9	118.88 (19)
C2—C1—H1C	109.5	C11—C10—C9	123.16 (19)
H1A—C1—H1C	109.5	C12—C11—C10	120.5 (2)
H1B—C1—H1C	109.5	C12—C11—H11	119.8
N1—C2—C3	114.78 (17)	C10—C11—H11	119.8
N1—C2—C1	124.35 (18)	C11—C12—C13	121.4 (2)
C3—C2—C1	120.87 (19)	C11—C12—H12	119.3
C4—C3—C8	117.15 (17)	C13—C12—H12	119.3
C4—C3—C2	122.31 (17)	C14—C13—C12	118.0 (2)
C8—C3—C2	120.54 (18)	C14—C13—C16	120.6 (2)
C3—C4—C5	121.51 (18)	C12—C13—C16	121.4 (2)
C3—C4—H4	119.2	C13—C14—C15	121.2 (2)
C5—C4—H4	119.2	C13—C14—H14	119.4
C6—C5—C4	120.5 (2)	C15—C14—H14	119.4
C6—C5—H5	119.8	C14—C15—C10	121.0 (2)
C4—C5—H5	119.8	C14—C15—H15	119.5
C5—C6—C7	118.56 (18)	C10—C15—H15	119.5
C5—C6—N2	119.34 (18)	C13—C16—H16A	109.5
C7—C6—N2	122.08 (18)	C13—C16—H16B	109.5
C8—C7—C6	120.26 (19)	H16A—C16—H16B	109.5
C8—C7—H7	119.9	C13—C16—H16C	109.5
C6—C7—H7	119.9	H16A—C16—H16C	109.5
C7—C8—C3	122.00 (19)	H16B—C16—H16C	109.5
C7—C8—H8	119.0		
			
O1—N1—C2—C3	178.84 (15)	C4—C3—C8—C7	−0.6 (3)
O1—N1—C2—C1	−0.6 (3)	C2—C3—C8—C7	179.64 (18)
N1—C2—C3—C4	−173.15 (18)	C6—N2—C9—C10	−176.97 (17)
C1—C2—C3—C4	6.3 (3)	N2—C9—C10—C15	−179.7 (2)
N1—C2—C3—C8	6.6 (3)	N2—C9—C10—C11	1.2 (3)
C1—C2—C3—C8	−173.93 (19)	C15—C10—C11—C12	−0.9 (3)
C8—C3—C4—C5	−0.3 (3)	C9—C10—C11—C12	178.23 (18)
C2—C3—C4—C5	179.52 (18)	C10—C11—C12—C13	0.0 (3)
C3—C4—C5—C6	1.6 (3)	C11—C12—C13—C14	0.7 (3)
C4—C5—C6—C7	−2.0 (3)	C11—C12—C13—C16	−179.1 (2)
C4—C5—C6—N2	179.47 (18)	C12—C13—C14—C15	−0.4 (3)
C9—N2—C6—C5	−132.4 (2)	C16—C13—C14—C15	179.4 (2)
C9—N2—C6—C7	49.1 (3)	C13—C14—C15—C10	−0.5 (3)
C5—C6—C7—C8	1.2 (3)	C11—C10—C15—C14	1.1 (3)
N2—C6—C7—C8	179.67 (18)	C9—C10—C15—C14	−178.03 (18)
C6—C7—C8—C3	0.1 (3)		

### Hydrogen-bond geometry (Å, °)

**Table:**

*D*—H···*A*	*D*—H	H···*A*	*D*···*A*	*D*—H···*A*
O1—H1···N2^i^	0.86 (1)	2.06 (1)	2.919 (2)	175 (3)

Symmetry codes: (i) *x*+1/2, −*y*+1/2, *z*−1/2.
